# Improving the local climate zone classification with building height, imperviousness, and machine learning for urban models

**DOI:** 10.1007/s43762-022-00046-x

**Published:** 2022-06-18

**Authors:** Kwun Yip Fung, Zong-Liang Yang, Dev Niyogi

**Affiliations:** 1 Department of Geological Sciences, Jackson School of Geosciences, The University of Texas at Austin, Austin, TX USA; 2 Department of Civil, Architectural, and Environmental Engineering, Cockrell School of Engineering, The University of Texas at Austin, Austin, TX USA

**Keywords:** Local Climate Zone, Machine Learning, Deep Learning, Urban Classification

## Abstract

The Local Climate Zone (LCZ) classification is already widely used in urban heat island and other climate studies. The current classification method does not incorporate crucial urban auxiliary GIS data on building height and imperviousness that could significantly improve urban-type LCZ classification utility as well as accuracy. This study utilized a hybrid GIS- and remote sensing imagery-based framework to systematically compare and evaluate different machine and deep learning methods. The Convolution Neural Network (CNN) classifier outperforms in terms of accuracy, but it requires multi-pixel input, which reduces the output’s spatial resolution and creates a tradeoff between accuracy and spatial resolution. The Random Forest (RF) classifier performs best among the single-pixel classifiers. This study also shows that incorporating building height dataset improves the accuracy of the high- and mid-rise classes in the RF classifiers, whereas an imperviousness dataset improves the low-rise classes. The single-pass forward permutation test reveals that both auxiliary datasets dominate the classification accuracy in the RF classifier, while near-infrared and thermal infrared are the dominating features in the CNN classifier. These findings show that the conventional LCZ classification framework used in the World Urban Database and Access Portal Tools (WUDAPT) can be improved by adopting building height and imperviousness information. This framework can be easily applied to different cities to generate LCZ maps for urban models.

## Introduction

Urbanization is defined as the process of the human population shifting from natural to artificial land surface (Tisdale 1942), including migration and clustering of the population from the rural to the urban area and transforming natural land cover to urban land use. Although urban areas cover less than 3% of the world’s land surface, the 2018 UN World Urbanization Prospect (United Nations 2019) shows that more than 55% of the global population resides on it. High population density (Oke 1973; Peng et al. 2012; Ramírez-Aguilar & Souza 2019), building materials (Arnfield 2003), anthropogenic activities (Peng et al. 2012; Shahmohamadi et al. 2011), and urban morphology (Oke 1981; Zhou et al. 2017) are some of the factors that create the warmer urban environment compared to the surrounding rural areas. This phenomenon is called urban heat island (UHI). UHIs have been found to exacerbate heatwaves (Founda & Santamouris 2017; Li & Bou-Zeid 2013; Zhao et al. 2018), precipitation (Changnon 1968; Fung et al. 2021; Liu and Niyogi 2019; Han et al. 2014), and air pollution (ozone concentration; Sarrat et al. 2006; Li et al. 2016; Swamy et al. 2017).

The evaluation of UHI impact can be performed by using land use land cover (LULC) data to separate the urban areas from the rural areas for measuring and simulating the temperature difference. The Moderate Resolution Imaging Spectroradiometer (MODIS; Friedl et al. 2010) and the Climate Change Institute Land Cover (CCI-LC) dataset developed by the European Space Agency (ESA) are examples of global LULC datasets with only urban class. The National Land Cover Database (NLCD) land cover data (Homer et al. 2011), only available in the US, contains four urban classes. The limited urban classes from these typical global and national LULC are often insufficient to represent the highly heterogeneous urban areas.

To address this deficiency, the Local Climate Zone (LCZ) classification was developed by Stewart and Oke (2012) and has ten urban LCZ classes (class 1–10) and seven natural LCZ classes (class A-G; Table 1). LCZs can be classified based on various properties, including building height, impervious surface fraction, canyon aspect ratio, surface albedo, anthropogenic heat flux, etc. Studies have shown that LCZs can capture the spatial variation of temperature (Stewart et al. 2014; Stewart & Oke 2012), ventilation (Zhao et al. 2020), and specific humidity (Yang et al. 2020). Several studies also showed that incorporating LCZ in numerical models, such as the Weather Research and Forecasting (WRF) model, can improve the simulated temperature (Molnár et al. 2019; Vuckovic et al. 2020) and precipitation (Patel et al. 2020) accuracy.

**Table 1:** The urban and natural LCZ classes (Stewart & Oke 2012)

Urban classes	Name	Natural classes	Name
1	Compact high-rise	A	Dense trees
2	Compact mid-rise	B	Scattered trees
3	Compact low-rise	C	Bush, scrub
4	Open high-rise	D	Low plants
5	Open mid-rise	E	Bare rock or paved
6	Open low-rise	F	Bare soil or sand
7	Lightweight low-rise	G	Water
8	Large low-rise		
9	Sparsely built		
10	Heavy Industry		

There are three major ways to generate an LCZ map (a) GIS-based, (b) remote sensing imagery-based, and (c) hybrid method (Lehnert et al. 2021). The GIS-based methods use the geometric properties derived from GIS databases such as buildings, roads, and topography to classify LCZs (Geletič & Lehnert 2016; Oliveria et al. 2020). Lehnert et al., (2021) has summarized the GIS-based method into Lelovics-Gál (Lelovics et al. 2014) and Geletič-Lehnert (Geletič & Lehnert 2016) method. Both methods calculate the geometric properties required for each class. In comparison, the Geletič-Lehnert method has an additional parameter related to the number of buildings per hectare. The primary disadvantage of the GIS-based method is data availability, because building information data may not be available for every city.

The remote sensing imagery-based method create an LCZ map using spectral properties retrieved from the satellite imageries. Such a methodology has been documented in the WUDAPT (Ching et al. 2018) project using Landsat 8 imageries (Bechtel et al. 2015). Landsat 8 imageries were first trimmed into the region of interest (ROI), and then different training areas were drawn using Google Earth. Both the Landsat 8 imageries and the training areas were then passed into a random forest classifier in the System for Automated Geoscientific Analysis (SAGA-GIS) software. Some studies have shown that implementing the deep learning classifiers, such as the convolution neural networks (CNNs), and different satellite imageries, such as Sentinel-2, can also improve the classification accuracy (Qiu et al. 2018; Yoo et al. 2019). Paticularly, Yoo et al. (2019) showed a 19–29% improvement in the urban classes when using the CNN classifier.

The hybrid methods make use of both GIS and remote sensing data to create the LCZ maps. The building height data are often used as auxiliary information to supplement remote sensing imageries. Yoo et al. (2020) have shown that local building height data derived from LiDAR can improve the classification accuracy of urban classes by 7–9%. Furthermore, Qiu et al. (2018) showed that additional global urban footprint, OpenStreetMap building layers, and Nighttime Light data could improve the accuracy of the urban-type classes even with a small sample size.

This study compared the LCZ classification performance from different machine/deep learning classifiers and urban auxiliary datasets. The study area and input features used in this study were described in section 2. The evaluation methodology and metrics were described in section 3. The comparison results of various classifiers were summarized in section 4.1, and the impacts and importance of including the urban auxiliary dataset were summarized in sections 4.2–4.5. The major findings and significance of the study were discussed in section 5. The hybrid framework used in this study can be used to advance the conventional classification framework suggested by WUDAPT and apply it to different cities for urban modeling studies.

## Study area and data processing

### Study area

**Fig. 1:**
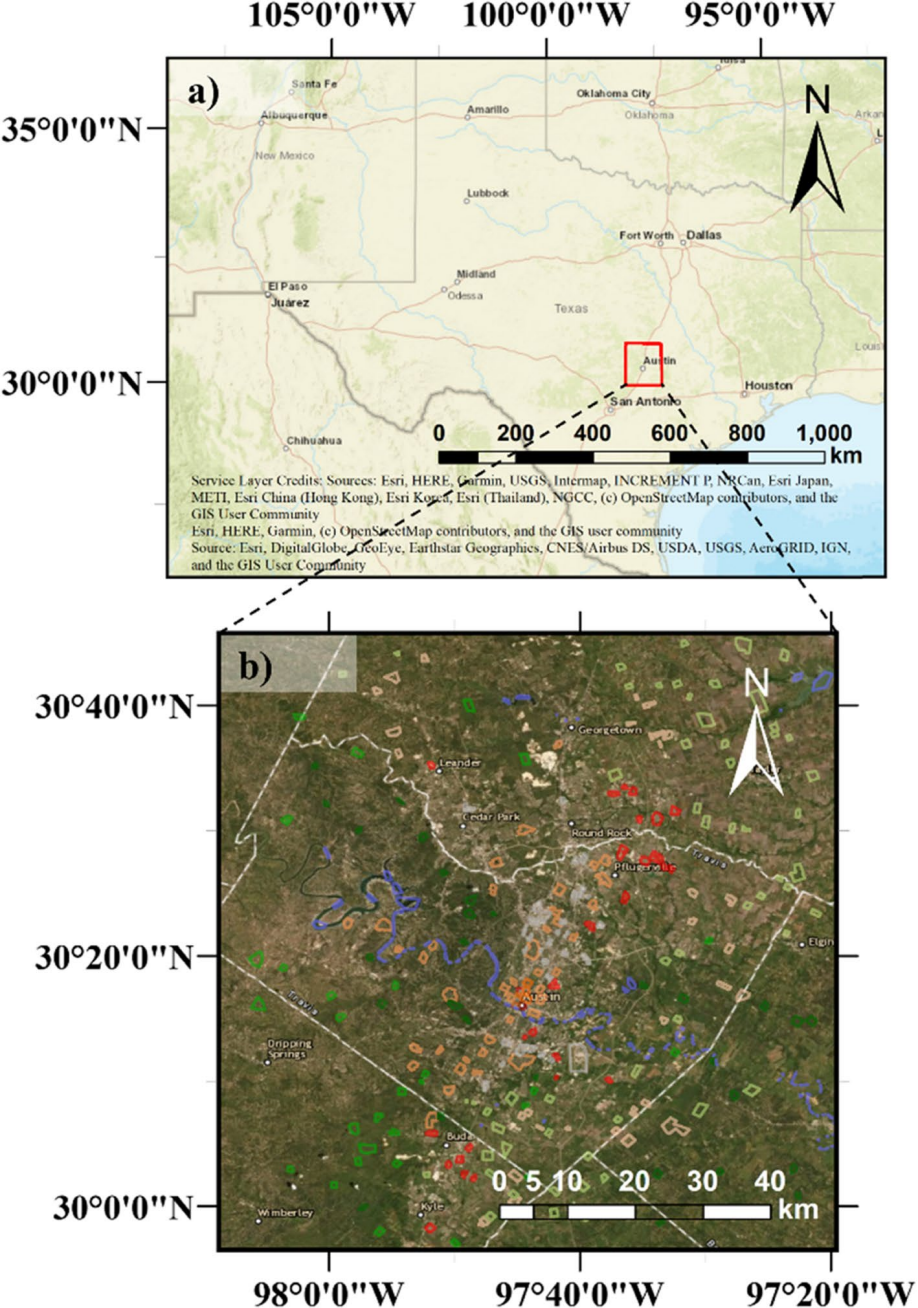
**a** The Texas map showing the Austin ROI (red box) investigated in this study. **b** The zoomed-in Austin map with training polygons for each LCZ class

Austin is located in central Texas (Fig. 1a) of the US and covers an area of 790 km^2^. It is the capital city of Texas, with a population of about 1 million, according to the 2020 census data (https://worldpopulationreview.com/us-cities/austin-tx-population). Austin is located in the transition zone between the dry desert on the west and the humid coastal region on the east. Under the Koppen Climate Classification, it is classified as a “humid subtropical climate” (Cfa). According to the Climate Summary report given by the National Weather Service, Austin’s winter (December – February) has a usual high temperature of 61 °F (16 °C) and precipitation of 6.64 in. (168 mm). The summer (June – August) has a typical high temperature of 90 °F (32 °C) and precipitation of 8.56 in. (217 mm). The annual-averaged rainfall is about 34.32 in. (872 mm), peaking in May, June, and October.

The ROI for this study has a latitude: 29.9 to 30.8^o^N and longitude: 97.3 to 98.1^o^W (Red box in Fig. 1a), which has embedded the Austin metropolitan area, including most of the Travis County and part of Williamson, Burnet, Hays, Bastrop, and Caldwell Counties. The ROI covers area with land use of residential, commercial, recreational, and other types of land uses, according to the Land Use Inventory map created by the City of Austin in 2018 (https://data.austintexas.gov/Locations-and-Maps/Land-Use-Inventory-Map/pstw-7bkg).

### Input features for LCZ classification

#### Landsat 8 imageries

Landsat 8 contains two instruments: Operational Land Imager (OLI) and Thermal Infrared Sensor (TIRS). OLI has nine spectral bands (wavelengths ranging from 0.43 μm to 2.29 μm). TIRS has two spectral bands (wavelengths ranging from 10.6 μm to 12.51 μm). All bands except band 8 (panchromatic) and band 9 (cirrus) were used as inputs following Bechtel et al. (2015) and Patel et al. (2020) to generate the 30-m Austin LCZs map. As seasonality strongly impacts vegetation spectral properties, we have included Landsat 8 imageries in different seasons. To minimize the impact of the cloud cover blockage, we ensure that all imageries do not have cloud cover on the ROI. Three Landsat 8 imageries were used (1 Nov 2018, 4 Jan 2019, and 26 Apr 2019). The Landsat 8 imageries were downloaded from the USGS EarthExplorer: https://earthexplorer.usgs.gov/

#### Lidar derived building height data

The building height data were provided by the City of Austin in 2017. The raw data provide different building footprint polygons in the format of shapefiles. Each footprint polygon has an attribute value for the average height derived from the Lidar Orthoimagery. The polygons were rasterized into 30-m spatial resolution. The data were downloaded from the City of Austin GIS Data Portal: https://austintexas.app.box.com/s/8ah8itbha7u6lis9eipypnz5ljvwta4t.

#### US national categorical mapping of building heights

The US national categorical mapping of building heights dataset (DOI: 10.5066/F7W09416) was derived from the Shuttle Radar Topography Mission (SRTM) data. SRTM data were acquired during February 11–22, 2000. The C-band Imaging Radar and X-band Synthetic Aperture Radar were equipped on the space shuttle. The antennas collected signals from the land surface at different angles to calculate the surface elevation. The SRTM data were processed and aggregated into different block groups according to census block groups. Data were only processed for the continental US. Each block group was classified into six classes and contains average height information. Data were downloaded from: https://www.sciencebase.gov/catalog/item/5775469ce4b07dd077c7088a.

#### Global man-made impervious surface data

The Global Man-made Impervious Surface (GMIS) dataset (Brown de Colstoun et al., 2017) was derived from the 2010 Global Land Survey Landsat image archive, which consists of Landsat 5 Thematic Mapper, Landsat 7 Enhanced Thematic Mapper, and Earth Observer 1 Advanced Land Imaging images. The GMIS dataset provides a 30-m resolution impervious percentage for each grid point globally. The data were downloaded from: https://sedac.ciesin.columbia.edu/data/set/ulandsat-gmis-v1.

#### National land cover database imperviousness data

The National Land Cover Database (NLCD) was developed by the US Geological Survey (USGS) for the past two decades. The imperviousness data were derived from Landsat images, IKONOS Space Imaging, and USGS National Aerial Photography Program Digital Orthophoto Quadrangles using a regression tree algorithm (Yang et al., 2003). The 2019 imperviousness data were available in 30-m resolution (Dewitz & U.S. Geological Survey, 2021). The data were downloaded from: https://www.mrlc.gov/data/nlcd-2019-percent-developed-imperviousness-conus.

## Methodology

This study extends the understanding of various machine learning classifiers and input data (Landsat 8 imageries, building height, and imperviousness) on the performance of urban-type classes using a similar framework as WUDAPT. This study used of the Texas Advanced Computer Center’s Stampede 2 supercomputer with one Indel Xeon Phi 7250 (“Knights Landing”) compute node with 68 cores, 1.4 GHz clock rate, and 96 Gb. of traditional Double Data Rate 4 (DDR4) Random Access Memory (RAM). We have tested four single-pixel classifiers, including K-Nearest Neighbor (KNN), Random Forest (RF), Gaussian Naïve Bayes (GNB), and Artificial Neural Network (ANN), which train and classify each pixel separately. In addition, a multi-pixel deep learning classifier CNN was also tested. CNN takes in the information of surrounding pixels for training and classification. The best performing single-pixel classifiers and CNN were used to further test the performance of different combinations of the input dataset. The setup consisted of four experiments. The first experiment ingested only Landsat 8 imageries (LS8), the second used only Landsat 8 and building height data (LS8 + BH), the third experiment used only Landsat 8 and imperviousness data (LS8 + GMIS), and the last one used all Landsat 8, building height, and imperviousness data (LS8 + BH + GMIS).

**Fig. 2:**
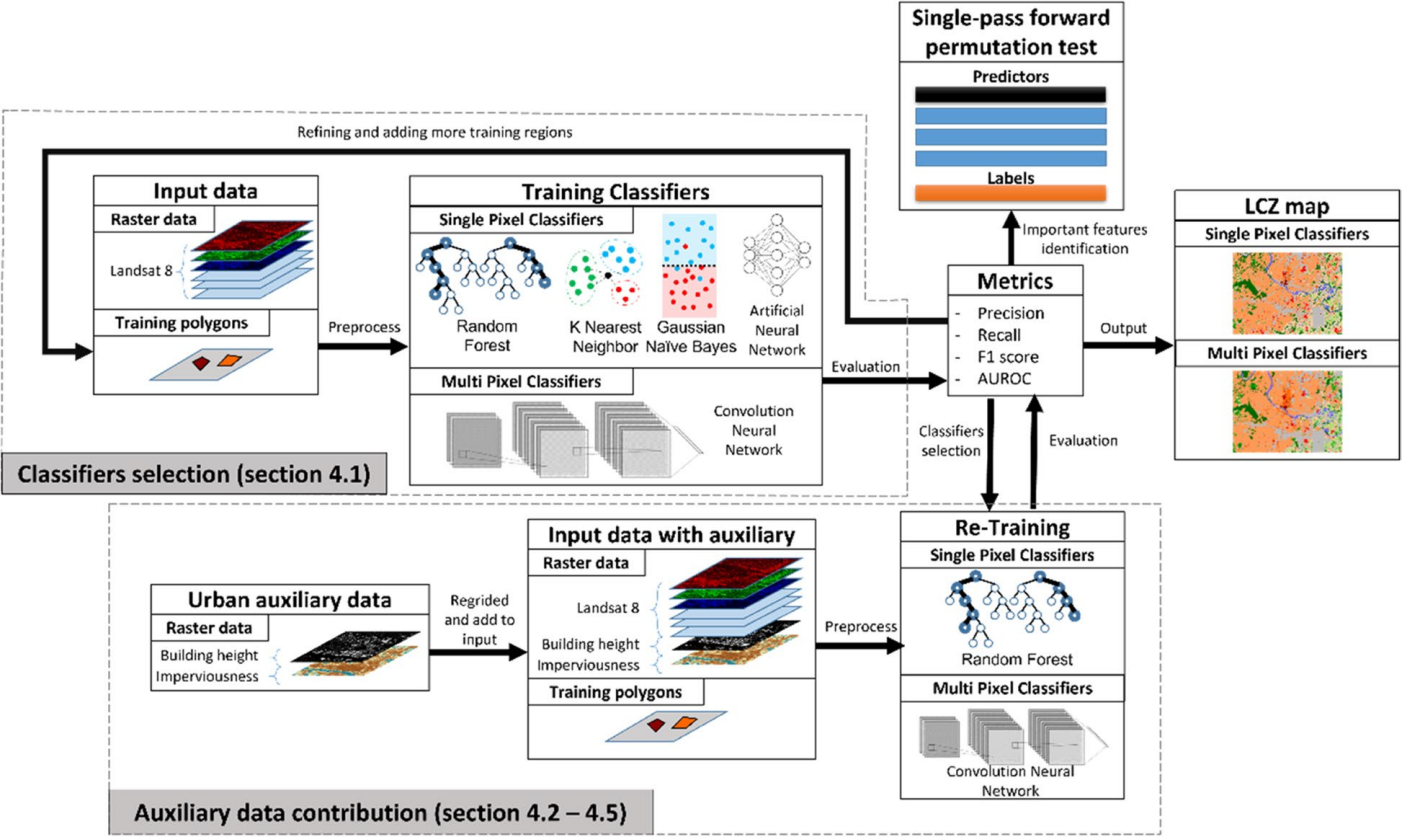
A workflow summary for this study

All input feature datasets (Landsat 8, building height, and imperviousness data) were reprojected and clipped into the ROI. To ensure that the dimensions of the input datasets were the same, the building height (BH) and imperviousness (GMIS) datasets were resampled or rasterized into the same grid system as Landsat 8 (LS8) imageries. Two BH datasets were tested, including Lidar derived dataset (BH_LD) and the US national categorical mapping of building height dataset (BH_C). The Lidar datasets are not always available, while the US national categorical dataset is available at the national level. Showing the value of using this SRTM-derived categorical building height dataset may help extend the training accuracy to cities with Lidar data available. Then training polygons were created using Google Earth (See section 3.1). The input features concerning the training pixels as identified by the training polygons were passed into different machine learning classifiers to train and evaluate the models. The optimal classifier was then determined using the LS8 imageries only, following the guidelines from WUDAPT. The identification of the optimal classifier was followed by the evaluation of the value of the BH_LD and GMIS. Then, multiple sources of urban auxiliary datasets were tested. Lastly, a single-pass permutation test was done to identify the prominent feature in the classifiers. The workflow is summarized in Fig. 2).

### Input features preprocessing

LCZ polygons were digitized in Google Earth by constructing polygons to bound a relatively homogeneous region for each class (Fig. 1b). There are eight urban-type classes in Austin (class 1–6, 8, and 9). Polygons of the same class should cover different variations, such as different roof colors, building materials, and building spacing. As suggested by WUDAPT, each class should have at least five polygons, and each polygon should have a size of at least 0.125 km^2^ (Stewart & Oke 2012). However, some classes (class 1, 2, and 4) in Austin were unavailable to follow this suggestion. The training polygons were applied to all features by extracting the corresponding regions of each feature as training and testing data.

There were altogether 411 polygons and 250,434 pixels labelled. 70% of the labelled pixels (175,303 pixels) were used in the training process for the single-pixel machine learning classifier. The rest of the pixels (75,131 pixels) were used to test the accuracy of the classification results. The 7:3 ratio of training:testing was kept for all classes. To avoid bias due to the combination of training and testing sample separation, each classifier was cross-validated 20 times using various training and testing samples. The ensemble of 20 times classification results was evaluated. Considering that some classes have fewer samples, those classes were upsampled to a similar number of samples by duplicating the samples multiple times.

**Fig. 3:**
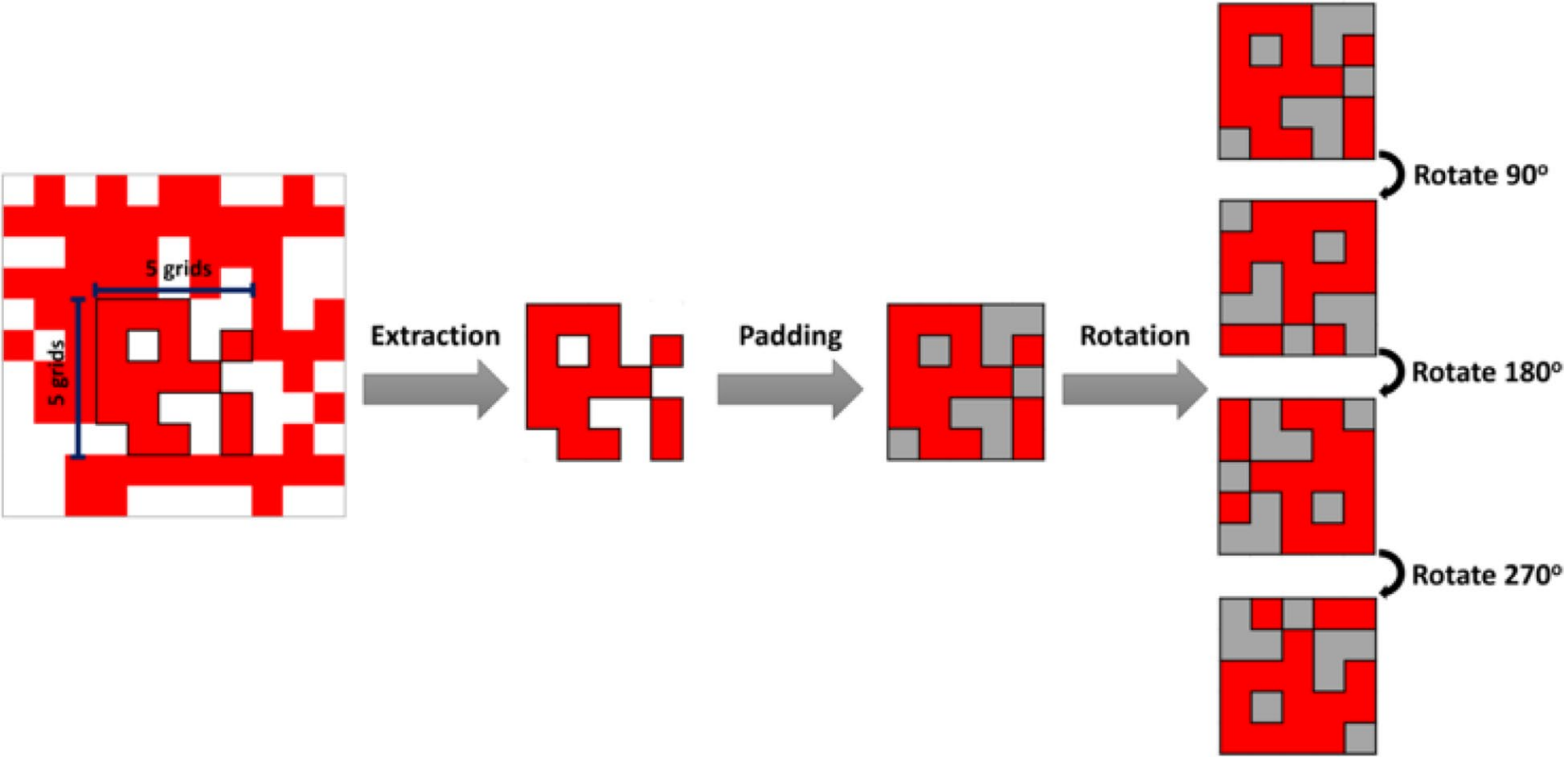
Framework for generating CNN inputs by rotating samples

The training polygons preprocessing processes for CNN are slightly different from the other single-pixel machine learning classifiers. First, the input features bounded by the polygons were extracted in the same way. Second, the extracted features were trimmed into patches of 5 by 5 pixels, and the surroundings were padded as zeros if the patches were not perfectly trimmed into 5 by 5. Third, the class with fewer patches were also upsampled. Fourth, these patches were separated into training and testing using the 7:3 ratio for 20 times with different training and testing splitting combinations. Then, each patch in both training and testing was rotated for 90^o^, 180^o^, and 270^o^ to improve the training dataset’s diversity without increasing the training samples (see Fig. 3).

The evaluation of the machine learning classifiers was first done using the Landsat 8 imageries only. Then, the best single-pixel classifier and CNN were selected to evaluate the value of including the building height and imperviousness dataset.

### Machine learning classifier selection

**Fig. 4:**
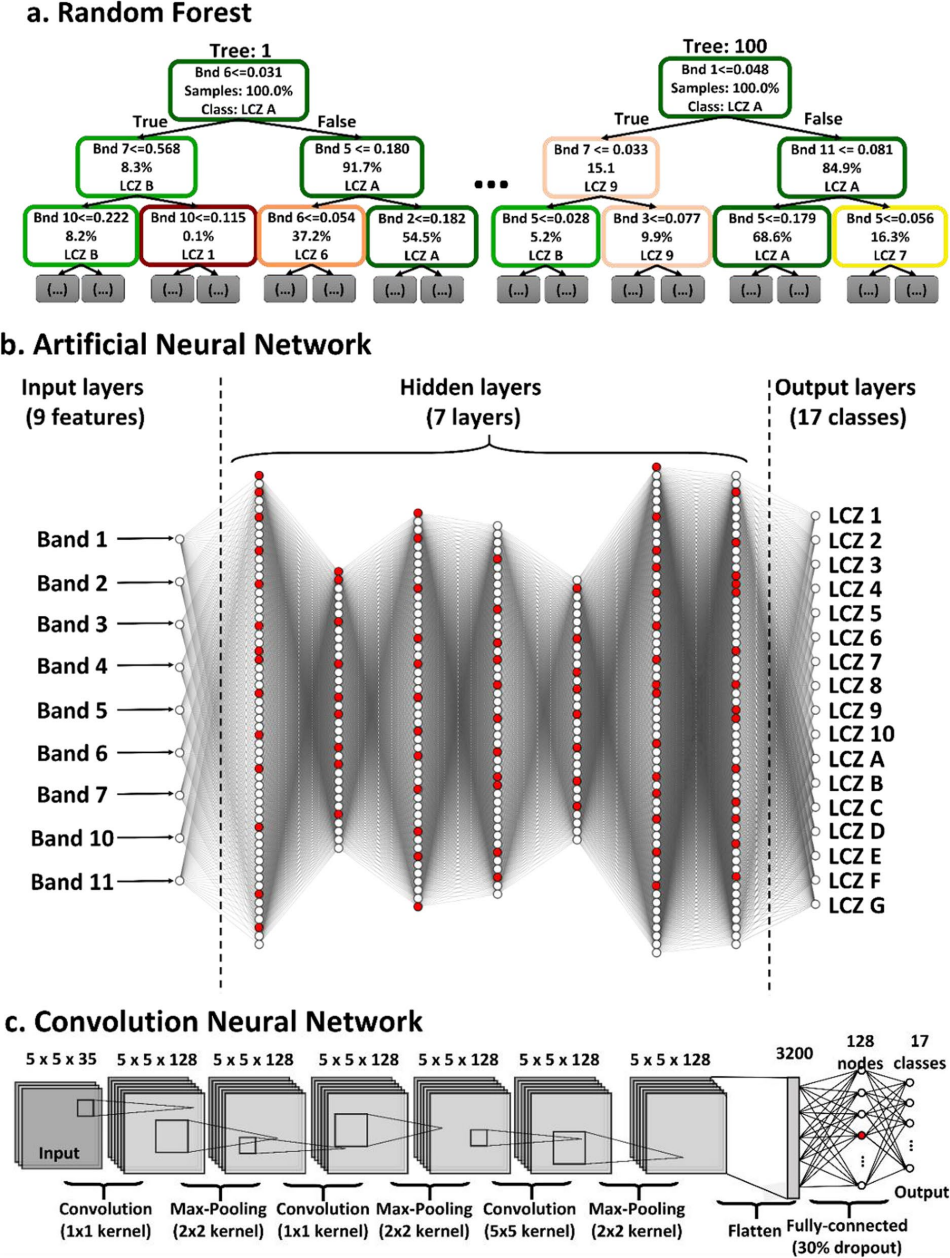
The architecture of **a** random forest (RF), **b** artificial neural network (ANN), and **c** convolution neural network (CNN) classifier used in this study. Red circles in ANN and CNN denote the dropout nodes

The optimal machine learning model was first selected by using the LS8 only. The machine learning classifiers tested include the single-pixel classifiers: K-Nearest Neighbor (KNN), Random Forest (RF), Gaussian Naïve Bayes (GNB), and Artificial Neural Network (ANN), and multi-pixel classifier: CNN. For the KNN classifier, 5, 10, and 15 nearest neighbor were tested. In addition, 10, 25, 50, and 100 trees were tested with the RF classifier. RF models (Fig. 4a) were trained with bootstrap samples, and the trees’ depths were expanded until all leaves were pure (i.e. children nodes contain only one result). GNB was tested by assuming the likelihoods are Gaussian.

Many more parameters need to be tested for the ANN classifier (Fig. 4b). The “Optuna” package in python was used to select the optimal combination of the activation functions (including rectified linear unit, sigmoid, softmax, softplus, softsign, hyperbolic tangent, scaled exponential linear unit, and exponential linear unit), number of layers (ranging from 2 to 10 hidden layers), and number of nodes (ranging from 12 to 66). To avoid overfitting, 25% of the nodes in each layer were randomly turned off. The selected ANN model configuration was as follows: hyperbolic tangent activation function, seven hidden layers, the numbers of nodes were 57, 34, 48, 45, 32, 59, and 57, respectively.

For the CNN classifier (Fig. 4c), four convolution layers with 5 × 5 kernel size and 128 nodes were included. The rectified linear unit was used as the activation function in each convolution layer. Then, a 2x2 max pooling was used with padding the output with zeros at the surrounding to maintain the output at a size of 5 × 5. A 25% dropout nodes was used in each convolution layer to avoid overfitting. Finally, the softmax activation function was used for the fully connected layer at the end to convert the output of maximum probability as the final class.

### Single-pass forward permutation test

The single-pass forward permutation test was used to evaluate the relative importance of the input feature in altering the outcome, following the idea introduced by Breiman (2001). The importance of the input feature was measured by evaluating the accuracy metrics reduction after random shuffling to cause a mismatch between the input features and the labels. Only one feature was shuffled each time. First, the classifier (*F*) was trained using the whole input features matrix (*X*) contains all features (*x*_*j*_). Second, the F1-score and the area under the receiver operating characteristics (ROC) curve (AUROC) were used as the evaluation metrics (*m*).

1 $$m=F(X)$$

The metrics were calculated as follows:

2 $$\mathrm{Precision}= TP/\left( TP+ FP\right)$$

3 $$\mathrm{Recall}= TP/\left( TP+ FN\right)$$

4 $$\mathrm{F}1\ \mathrm{score}={\left( Recal{l}^{-1}+ Precisio{n}^{-1}\right)}^{-1/2}$$

5 $$\mathrm{True}\ \mathrm{positive}\ \mathrm{rate}\ \left(\mathrm{TPR}\right)= TP/\left( TP+ FN\right)$$

6 $$\mathrm{False}\ \mathrm{positive}\ \mathrm{rate}\ \left(\mathrm{FPR}\right)= FP/\left( FP+ TN\right)$$

where TP is true positive, FP is false positive, TN is true negative, and FN is false negative. A ROC curve is constructed by the relationship of TPR (eq. 5) against FPR (eq. 6) for a classifier with various parameters. AUROC has a range from 0.5 to 1.0, which 1.0 indicates the classifier has good performance. Third, each feature (*x*_*j*_) was shuffled each time to create a permuted input feature matrix (${X}_j^{perm}$), and the new evaluation metrics were calculated (${m}_j^{perm}$; eq. 7). The metric losses (*L*_*j*_; eq. 8) were calculated and ranked to evaluate the importance of each feature (*x*_*j*_).

7 $${m}_j^{perm}=F\left({X}_j^{perm}\right)$$

8 $${L}_j=m-{m}_j^{perm}$$

## Results

### Performance of machine learning classifiers

The KNN, RF, GNB, and ANN classifiers carry out training and classification for every pixel individually. The CNN takes in 5 x 5 pixels and produces output at a spatial resolution of twenty-five times coarser than the input. Therefore, we have separated the classifiers into two different sets: single-pixel and multi-pixel classifiers. The best model in single-pixel and CNN classifiers were selected. Multiple indices were used to evaluate the classification accuracy performance: precision, recall, F1-score, and AUROC.

Each index can be calculated as either micro- or macro-average. Every individual sample was weighted the same in the micro-average method, while the macro-average method weighted every class equally. Therefore, the sample imbalance between classes does not affect macro-average indices. Austin has a small commercial center, which makes it impossible to create many training samples for the compact high-rise and compact mid-rise classes compared to the open low-rise class. Hence, sample imbalance is an issue, and macro-average indices should be considered when there are inconsistent results with micro-average indices.

**Fig. 5:**
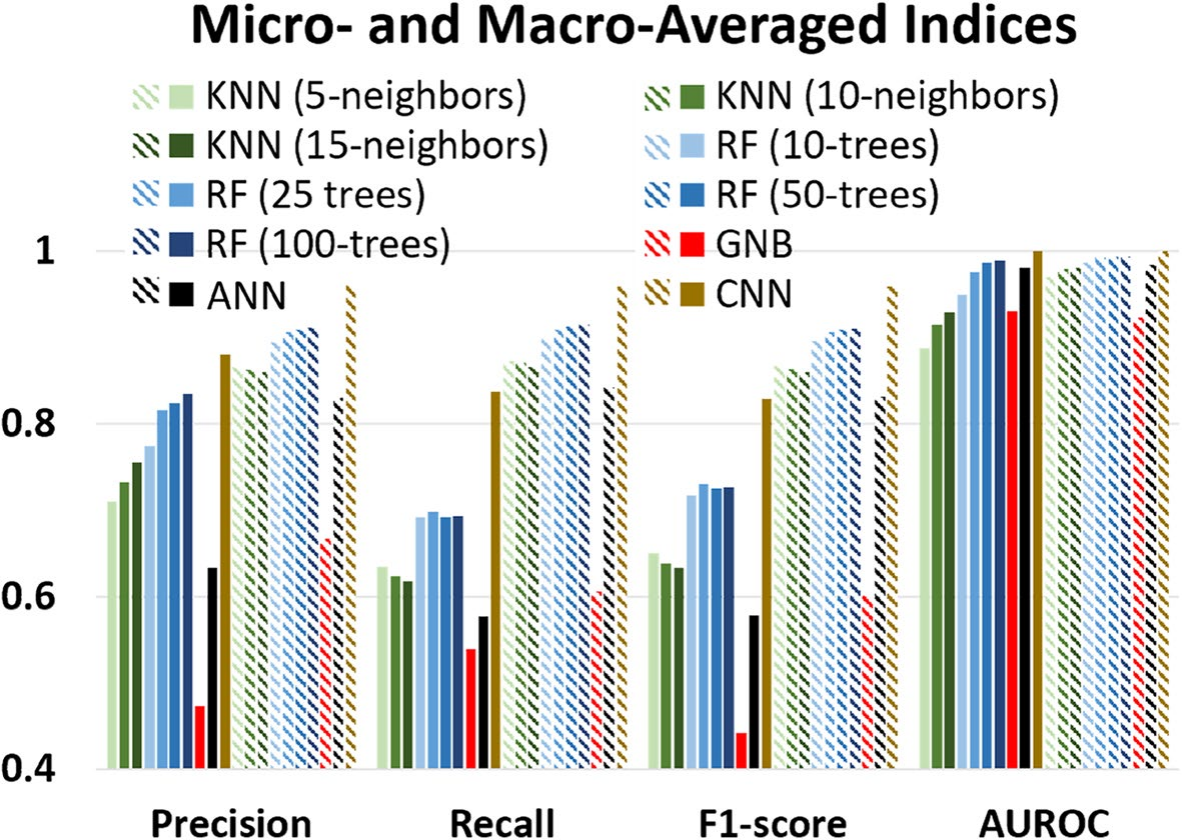
The evaluation indices (macro- and micro-average of precision, recall, F1-score, and AUROC) for different machine learning classifiers. Solid (stripe) shading indicates the macro-(micro-) averaged indices

Fig. 5 summarizes the indices of different machine learning classifiers using only the LS8 inputs. Within the KNN experiments, the macro-averaged (solid green bars) recall and F1-score show that 5-neighbors (KNN5) has better performance. In contrast, the macro-averaged precision and AUROC show that 15-neighbors (KNN15) performed the best, not giving a consistent result about the best KNN classifier. All micro-averaged indices (stripe green bars) show that 5, 10, and 15-neighbors KNN did not significantly differ from each other (lee than 0.01 difference). In the RF experiment, both macro-averaged (solid blue) and micro-averaged (stripe blue) indices also show that increasing the ensemble trees from 10 to 25 gives the greatest improvements (Precision: 0.041, Recall: 0.074, F1-score: 0.013, AUROC: 0.027). The improvement from 25 ensemble trees to 100 trees was weaker (Precision: 0.019, Recall: −0.005, F1-score: -0.004, AUROC: 0.013). Overall, the greater number of ensemble trees still performed better with higher AUROC. GNB performs better when the classes are well-separated. However, urban classes are often not well-separated due to the similar spectral properties between classes, such as roof colors. Therefore, the GNB model (red bars in Fig. 5) performed the worst among all models. Overall, the RF classifiers with 100 ensemble trees (RF100) performed the best among the single-pixel classifiers, with F1-score and AUROC 15% and 1% higher than KNN15, respectively. The only multi-pixel classifier used in this was CNN (brown bars in Fig. 5), which performed significantly better than RF100 in all indices, F1-score, and AUROC are 14% and 1% higher than RF100, respectively.

The computational time for each algorithm was summarized in Table 2. The RF classifiers take less time (less than 30 minutes) than the KNN and ANN classifiers for the whole classification process. Although GNB used the least time for classification, the accuracy is too low compared with other classifiers. The CNN algorithm takes most of the time in the preprocessing stage when trimming samples into patches and rotating samples occurs, while the classification and training time is about 10 minutes. Therefore, the RF and CNN classifiers were selected for further analysis.

**Table 2:** The computational efficiency for different machine learning algorithms in preprocessing, training, validating, and classifying

Classifiers	Preprocess (s)	Training time (s)	Validation time (s)	Classification time (s)
KNN-5	590.7	303.1	685.5	>100,000
KNN-10	590.7	280.5	784.8	>100,000
KNN-15	590.7	289.2	846.7	>100,000
RF-10	590.7	65.4	2.7	46.1
RF-25	590.7	162.3	4.8	112.3
RF-50	590.7	323.7	8.3	217.8
RF-100	590.7	660.8	15.3	432.3
GNB	590.7	1.0	3.5	58.3
ANN	590.7	937.1	18.7	973.0
CNN	10,469.0	532.6	7.7	83.8

### Performance of different datasets in RF100

**Fig. 6:**
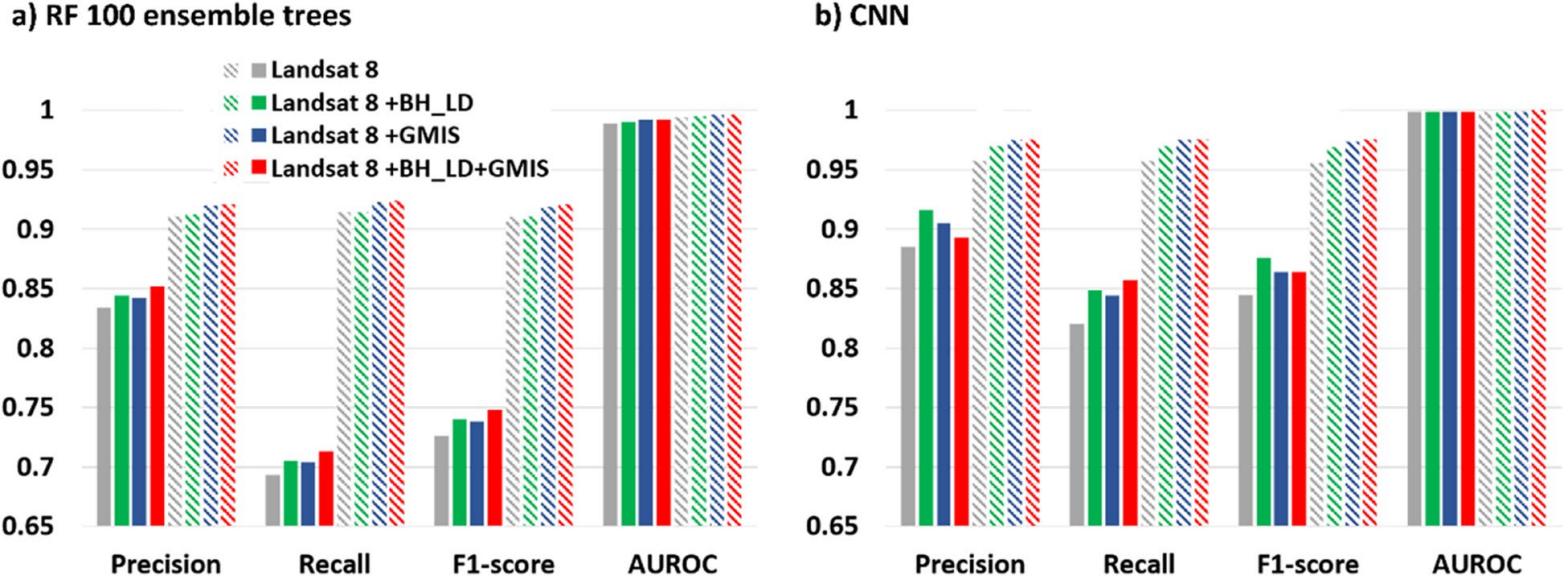
The evaluation indices (macro- and micro-average of precision, recall, F1-score, and AUROC) for different input datasets using **a** RF100 and **b** CNN classifier. Solid (stripe) shading indicates the macro-(micro-) averaged indices

**Fig. 7:**
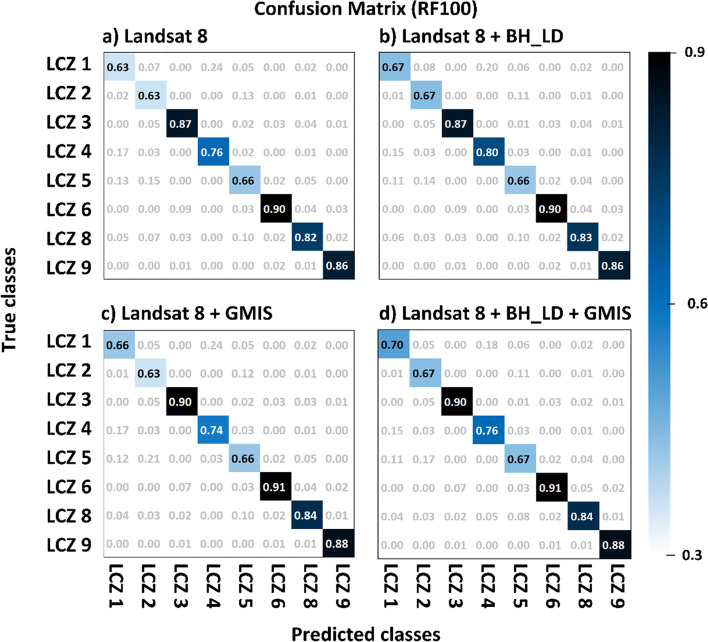
The confusion matrix of the urban-type classes from RF100 classifier using **a** LS8, **b** LS8 + BH_LD, **c** LS8 + GMIS, and **d** LS8 + BH_LD + GMIS input dataset

After selecting the RF100 and the CNN classifiers, the effect of urban auxiliary datasets (BH_LD and GMIS) were evaluated (Fig. 6). Considering the random forest classifier (Fig. 6a), the precision, recall, F1-score, and AUROC also show a higher score after adding either urban auxiliary datasets or both as the input features. The macro-averaged indices show that including building height has slightly more improvement (F1-score: 1.93%; AUROC: 0.101%) than imperviousness (F1-score: 0.985%; AUROC: 0.101%). While the micro-average indices show including imperviousness feature has more significant improvement (F1-score: 0.110%; AUROC: 0.101%) than the building height feature(F1-score: 0.989%; AUROC: 0.201%) dataset. The results show that including both urban auxiliary datasets have the best performance with the macro-averaged F1-score and AUROC improvement of 3% and 0.3%, respectively, compared with using Landsat 8 as the only input feature. This can be explained by the confusion matrix normalized by predicted labels (Fig. 7).

**Fig. 8:**
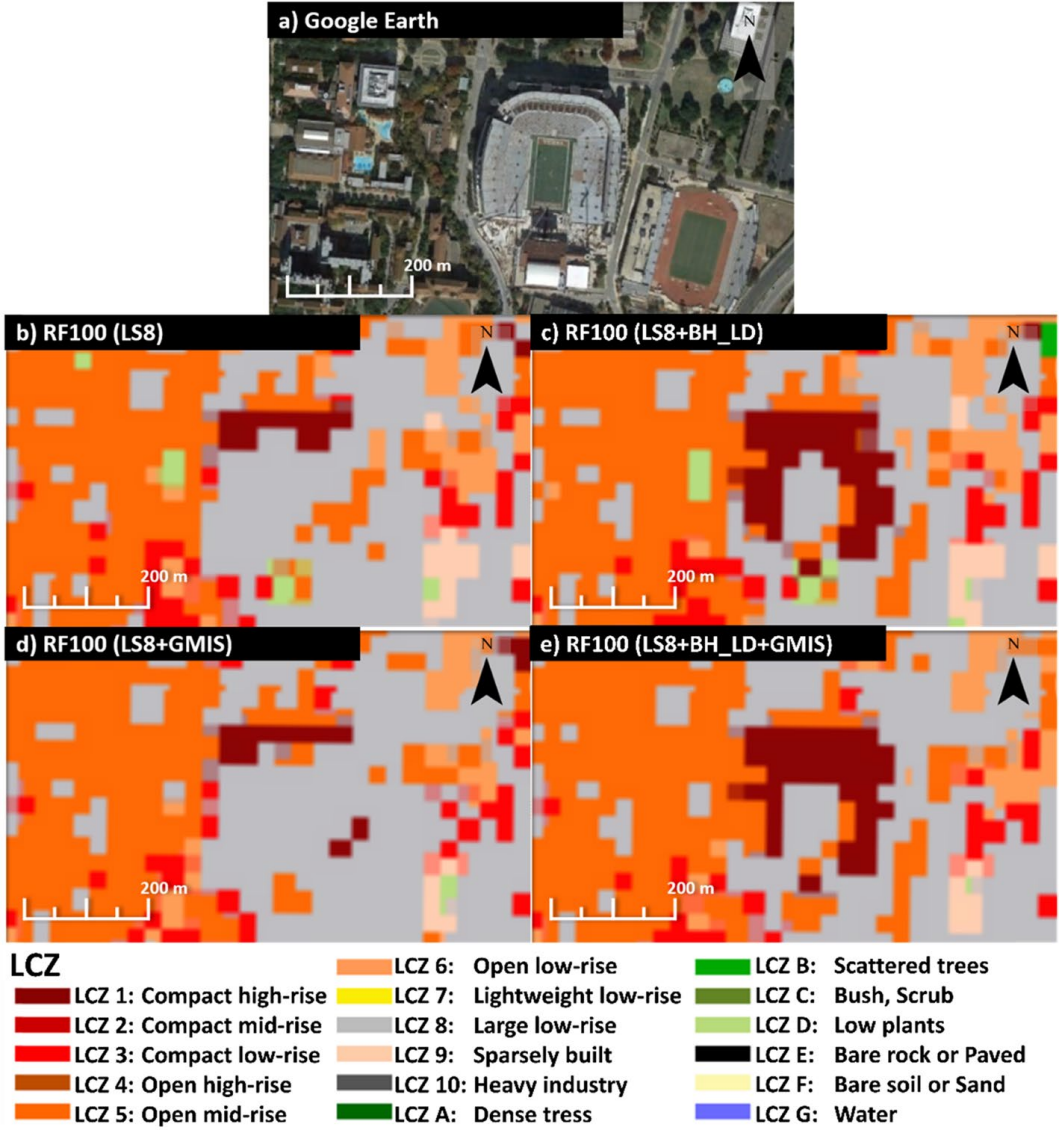
The **a** Google Earth image, **b** RF100 with LS8 as input, **c** RF100 with LS8 and BH_LD as input, and **d** RF100 with LS8, BH_LD, and GMIS as input at the Texas Memorial Stadium

A significant improvement of the building height dataset was found in high-rise urban classes. LCZ 1, 2, and 4 also experienced a 0.04 increment of accuracy (see Fig. 7a and b). Figure 8 shows an example of the Texas Memorial Stadium that was misclassified as the large low-rise (class 8) when using LS8 only as the input feature (Fig. 8b) or LS8 + GMIS (Fig. 8d). However, by including the building height dataset (Fig. 8c and 5e), the stadium was regarded as mid- and high-rise class. The mid- and high-rise buildings have highly variable building heights. Therefore building height data can provide extra vertical information to distinguish mid- and high-rise from the low-rise.

**Fig. 9:**
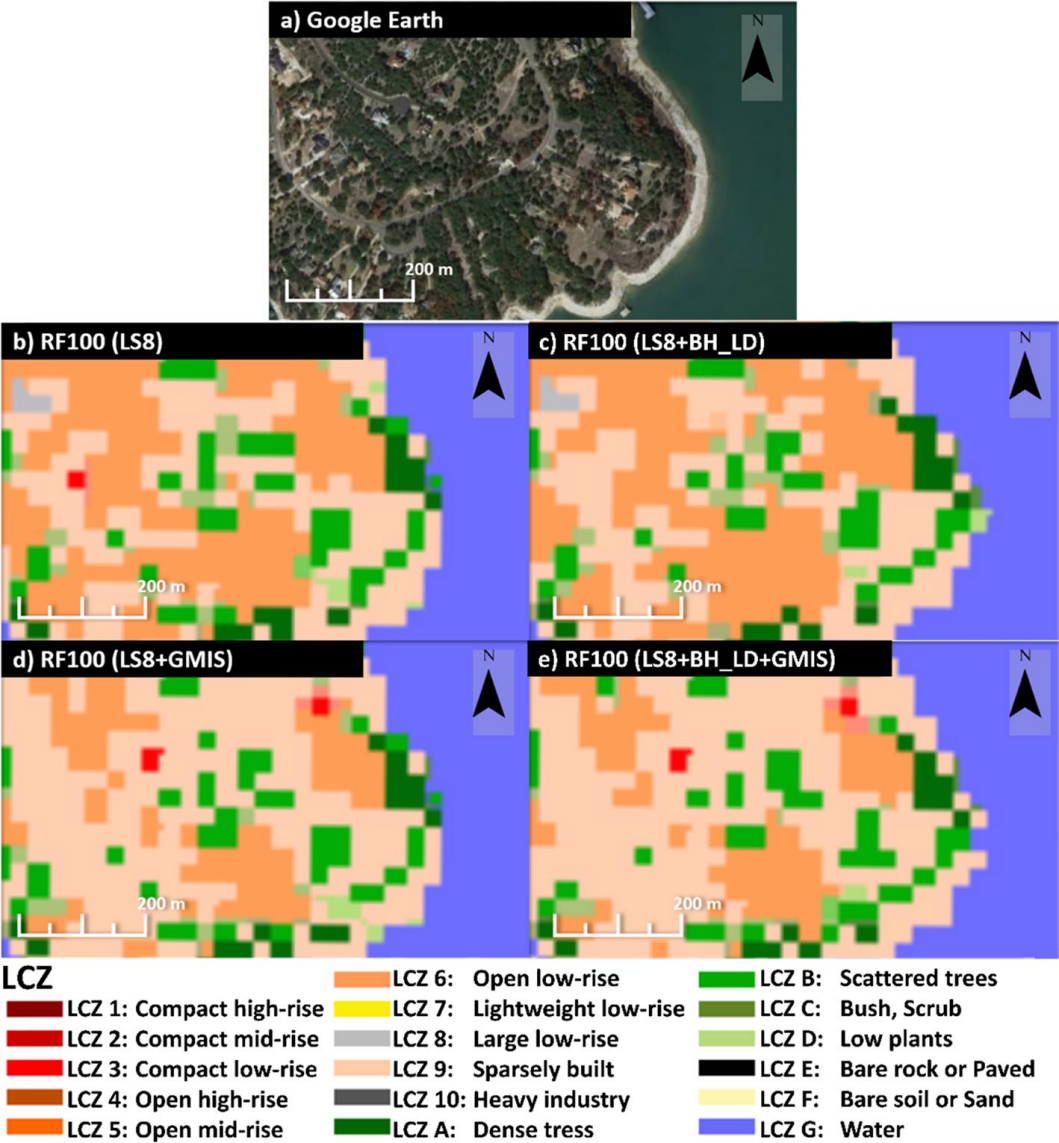
The **a** Google Earth image, **b** RF100 with LS8 as input, **c** RF100 with LS8 and BH_LD as input, and **d** RF100 with LS8, BH_LD, and GMIS as input at the Northlake Hills

On the other hand, imperviousness feature improves low-rise classes. Class 6, 8, and 9 also had 0.01–0.02 accuracy improvement (see Fig. 7a and Fig. 6c). Figure 9 shows an example at the Northlake Hill, LS8 (Fig. 9b) and LS8 + BH_LD (Fig. 9c) experiments misclassified the sparely built class (class 9) into open low-rise (class 6), while LS8 + GMIS (Fig. 9d) and LS8 + BH_LD + GMIS (Fig. 9e) experiments captured most sparsely built class (class 9). The low-rise classes have much more uniform building height, making the BH_LD dataset indistinguishable. GMIS data can provide extra information for the impervious materials coverage to differentiate between open and more compact classes.

Hence, the LS8 + BH_LD + GMIS experiment performs best in all indices (Red; Fig. 6a). The confusion matrixes show that LS8 + BH_LD + GMIS (Fig. 7d) could inherit most of the advances brought by the building height and imperviousness datasets. The performance of class 1, 2, 5, 6, 8, and 9 also improved compared to the conventional LS8 only (Fig. 7a). However, class 1 and 2 performed poorer than the other classes due to less training and testing samples (Fig. 7a). The macro-average indices weigh each class equally. Hence, the poor performance of class 1 and 2 weighs more in the macro-average indices than the micro-averaged indices. Therefore, macro-averaged indices were comparatively lower than micro-averaged indices. At the same time, the smaller sample size for class 1 and 2 could lead to a more substantial improvement due to building height than the imperviousness feature shown in Fig. 6a.

### Performance of different datasets in CNN

**Fig. 10:**
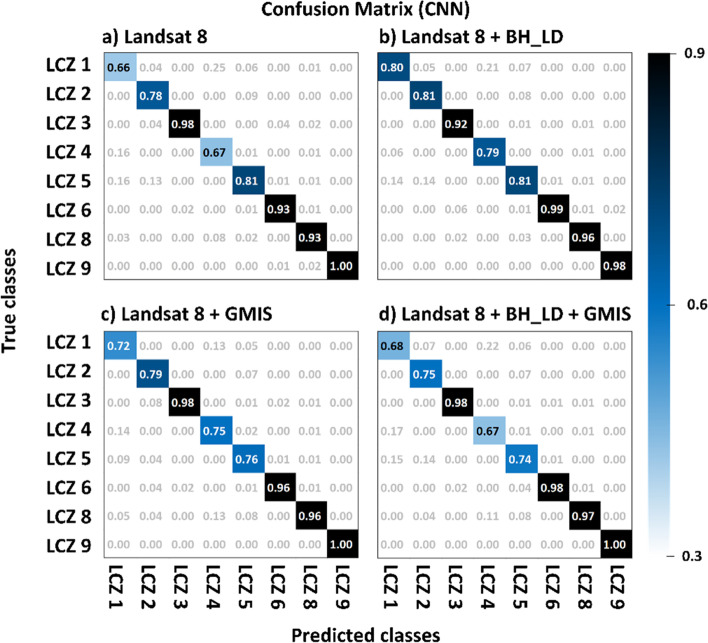
The confusion matrix of the urban-type classes from the CNN classifier using **a** LS8, **b** LS8 + BH_LD, **c** LS8 + GMIS, and **d** LS8 + BH_LD + GMIS input dataset

The macro-averaged (Fig. 6b solid shading) and micro-averaged indices (Fig. 6b stripe shading) also show that building height and imperviousness data could improve the classification performance. In addition, the macro-averaged show that building height data could offer a more significant performance improvement than the imperviousness data. This feature can be noted in the substantial magnitude of improvement for class 1 including building height feature, with a 0.14 increment (see Fig. 10b). At the same time, LS8 + GMIS (Fig. 10c) and LS8 + BH_LD + GMIS (Fig. 10d) only improves by 0.04 and 0.02, respectively. At the same time, the number of samples for class 1 was the least among all LCZs. Therefore, the building height feature only improves significantly more than the imperviousness feature in the macro-indices.

**Fig. 11:**
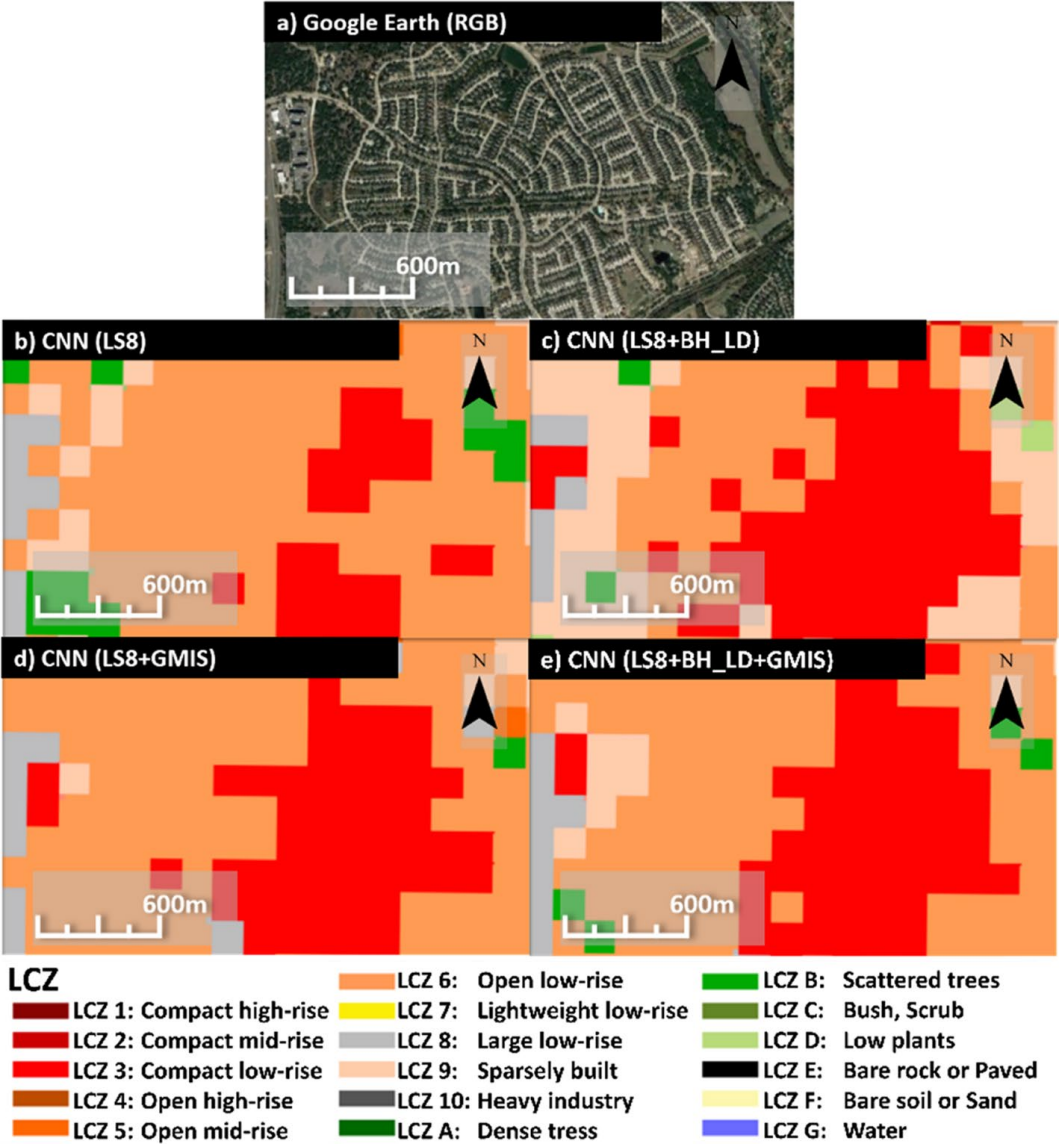
The **a** Google Earth image, **b** CNN with LS8 as input, **c** CNN with LS8 and BH_LD as input, and **d** CNN with LS8, BH_LD, and GMIS as input at the eastern Cedar Park

Figure 11 show a class 3 (compact low-rise) region in eastern Cedar Park, LS8 (Fig. 11b), which cannot capture the compactness and misclassify into class 6 (open low-rise). While including either or both building height and imperviousness data can also help capture the compactness. Compared with RF100, BH_LD (GMIS) dataset improves high-rise (low-rise) classes in CNN as CNN has convolution layers that take multi-pixels information for a single sample. Therefore, the compactness information was contained even when only considering the building height dataset.

The macro- and micro-indices did not show consensus about the choices of different dataset combinations to increase accuracy. Therefore, the only robust conclusion that can be made is that the classification accuracy can be improved by including either one or both the urban auxiliary datasets (BH_LD and GMIS).

**Fig. 12:**
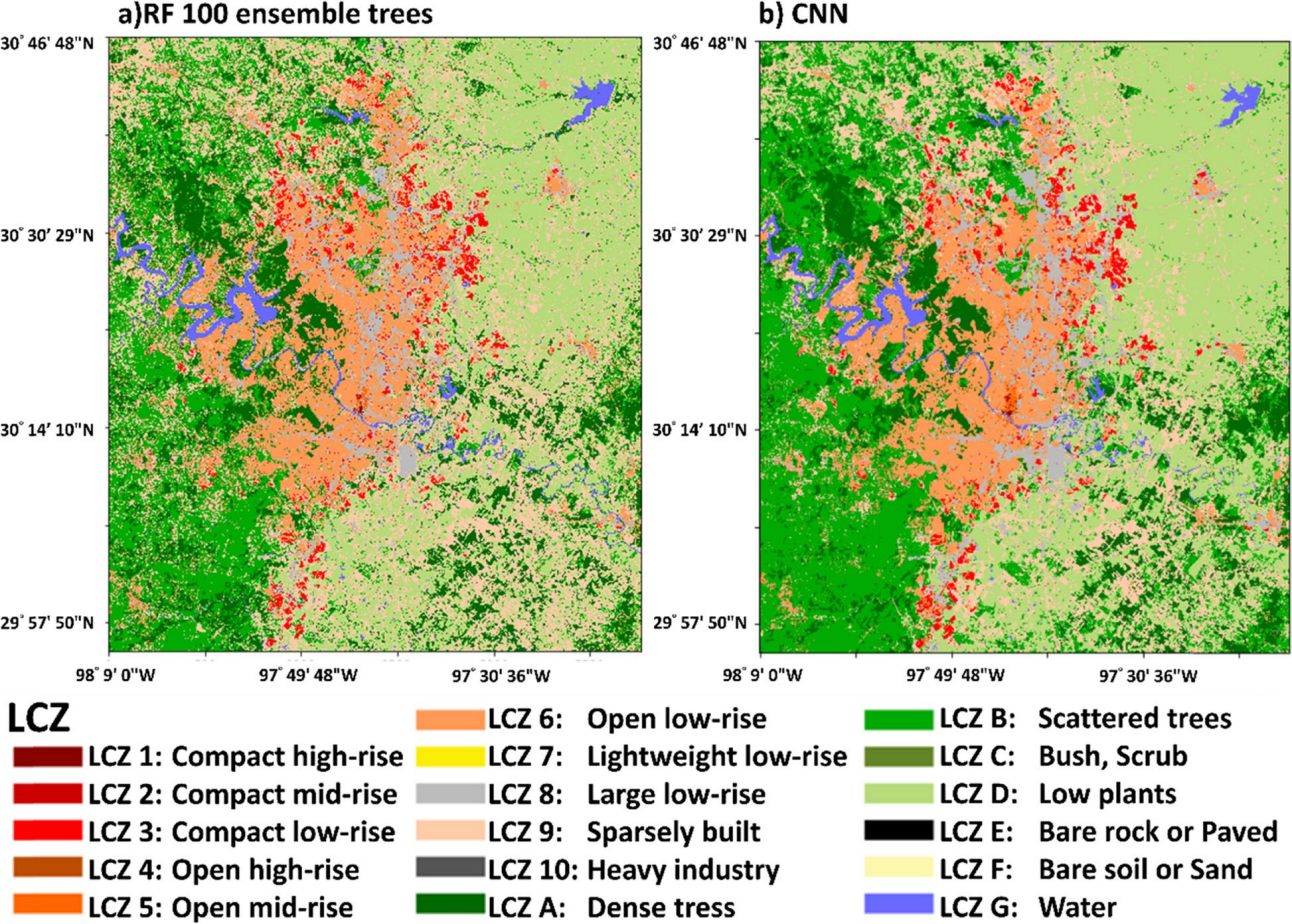
The LCZ products derived from **a** RF100 and **b** CNN classifier using LS8 + BH_LD + GMIS dataset

**Fig. 13:**
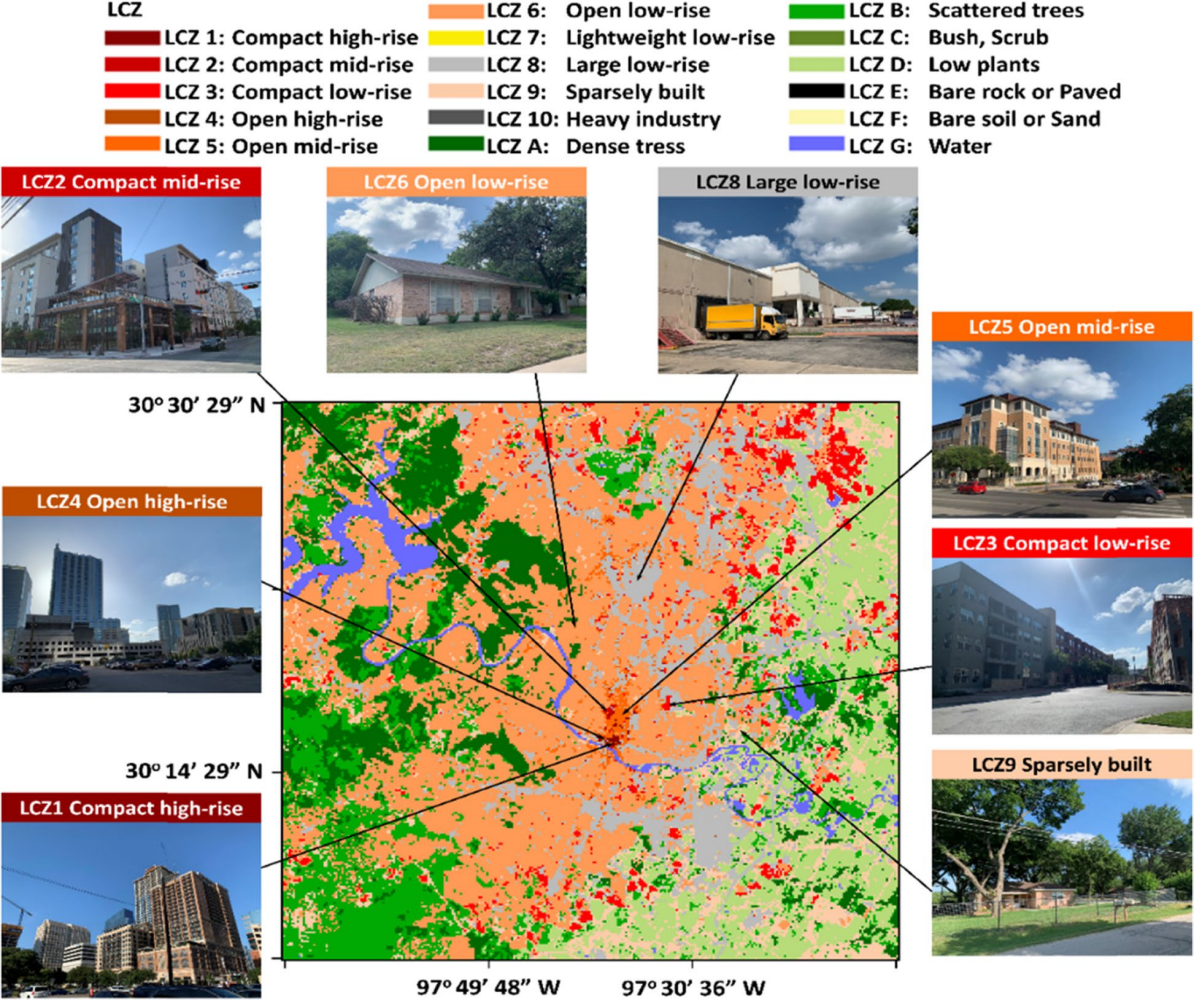
The ground-level photos of each urban LCZ class in Austin

The product of the LCZs map for Austin using LS8 imageries with building height and imperviousness with RF100 and CNN classifiers are shown in Fig. 12. Also, Fig. 13 shows the ground-level photos for each class in Austin, which can help validating the accuracy of classification results. Austin only contains urban class 1–6, 8, and 9. The high-rise classes (class 1 and 4) were found in the downtown area, located north of the Colorado River. The mid-rise classes (class 2 and 5) were situated north of the downtown area. The low-rise classes (class 3 and 6) were the dominant classes, which spread across Austin. The large low-rise class (class 8) was found at the sideway of the main roads. The sparsely built class (class 9) was found at the periphery of Austin. The coverage of each urban class from each classifier is summarized in Table 3. Both classifiers also show that Austin is covered mainly by urban class 6 (~ 30%), 8 (~ 10%), and 9 (~ 40–50%).

**Table 3:** Percentage coverage of urban LCZ classes in Austin classified by RF 100-trees and CNN

Urban classes	Coverage (RF 100-trees) (%)	Coverage (CNN) (%)
1	0.02	0.04
2	0.04	0.07
3	4.79	6.12
4	0.01	0.02
5	0.33	0.80
6	33.52	35.63
8	10.38	14.00
9	50.91	43.33

### Performance comparison with the LCZ Generator

**Fig. 14:**
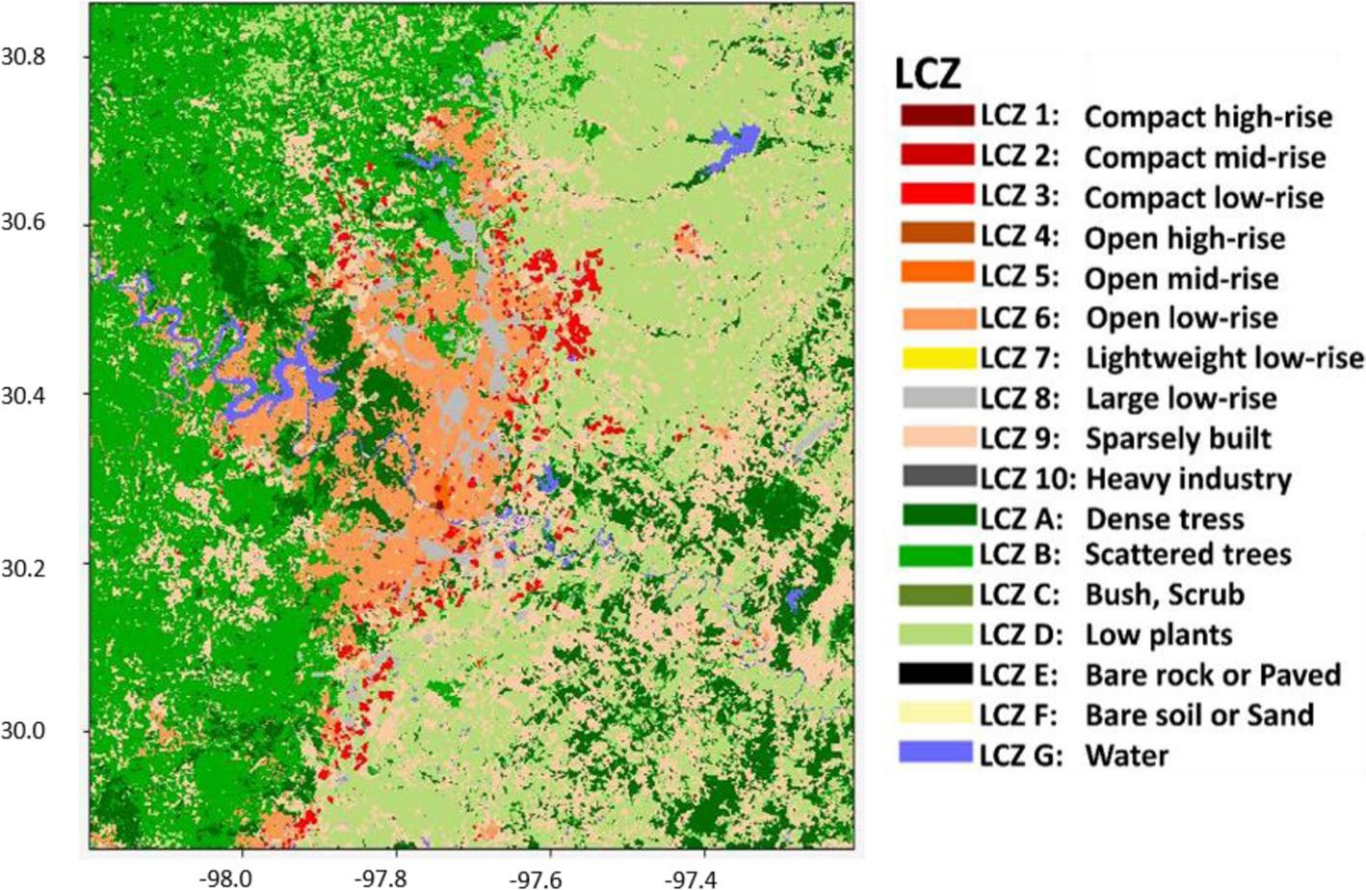
The LCZ map derived from LCZ Generator

The LCZ Generator developed by Demuzere et al. (2021) has the advantage of generating LCZ maps rapidly and easily. Users are only required to pass the training polygons into the generator, and the LCZ map will be generated in about 20 minutes. We compared the results from the LCZ Generator with this study. The identical training polygons used in this study were passed into the generator, and the product was shown in Fig. 14 (Fung 2022). The map generated by both processes is similar, at the center of Austin. However, the LCZ Generator misclassified some regions of open low-rise into sparely built at the periphery in southwestern Austin (see Figs. 12 and 14). The overall accuracy was used as the identical metric for both products, which is defined as the sum of true positives and true negatives divided by the number of samples. The accuracy for all classes from the LCZ Generator is 0.81 and 0.82 for urban classes, while the random forest classifiers using both urban auxiliary datasets in this study is 0.92 and 0.88, respectively. In conclusion, the methodology in this study has the advantage of accuracy and flexibility for incorporating urban auxiliary datasets. In contrast, the LCZ generator has the advantage of quickly and easily generating an LCZ map.

### Performance of different building height dataset

**Fig. 15:**
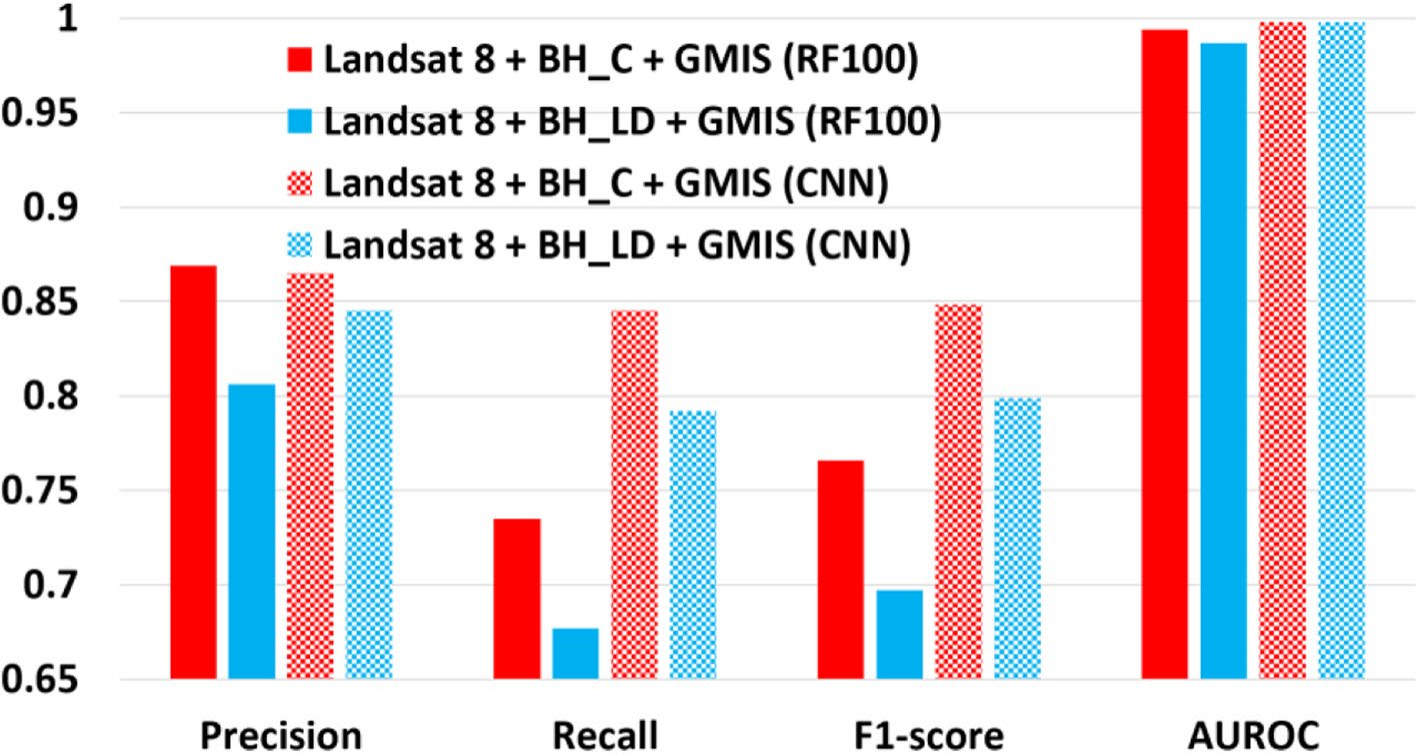
The macro indices (precision, recall, F1-score, and AUROC) for different BH datasets, BH_LD (red) and BH_C (blue) using RF100 (solid fill) and CNN (checked fill) classifier

As the availability of building height lidar (BH_LD) data is limited across the world and the US. The BH_C was tested to evaluate its value in improving classification results. Figure 15 shows that BH_C has a slightly higher value in all evaluation metrics (precision, recall, F1-score, and AUROC) than BH_LD. This confirms the importance of BH_C in improving the classification accuracy even building height lidar data are unavailable.

### Performance of different imperviousness dataset

**Fig. 16:**
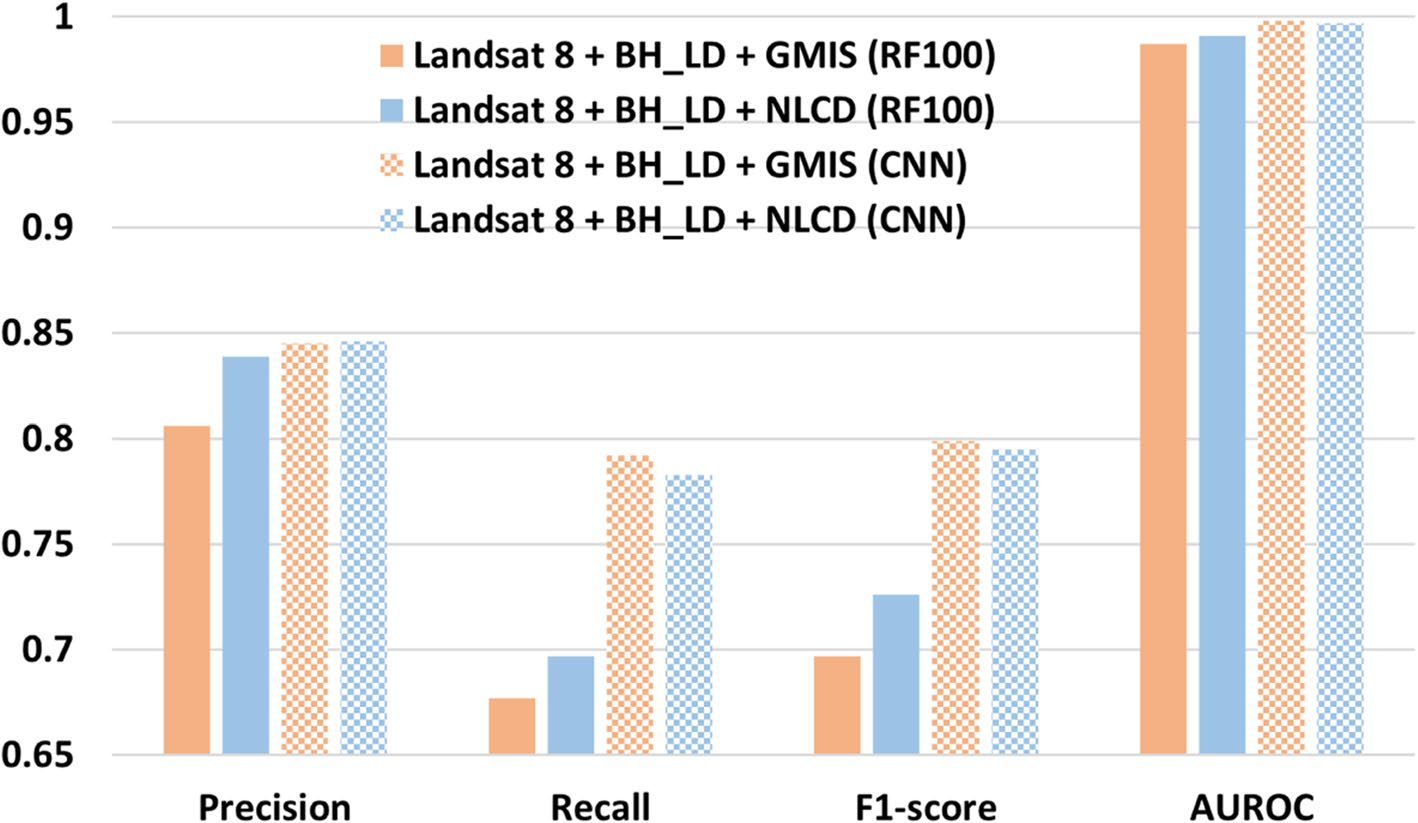
The macro indices (precision, recall, F1-score, and AUROC) for different imperviousness datasets, GMIS (orange) and NLCD (blue) using RF100 (solid fill) and CNN (checked fill) classifier

The most recent global imperviousness dataset GMIS used in this study is limited to 2010. Therefore, a more recent US national dataset NLCD in 2019 was tested with 20 cross-validation results. Figure 16 shows that NLCD has a slightly higher value in all evaluation metrics (precision, recall, F1-score, and AUROC) for the random forest classifier, while the impact on the CNN classifier is insignificant. This confirms that the more recent imperviousness can improve classification accuracy. However, the NLCD dataset is only available in the US. Based on the location of interest, including the GMIS dataset can still provide a significant improvement for cities located out of the US.

### Importance of input features

The single-pass forward permutation test permutes one feature at a time to see the F1 score and AUROC decline. The more significant decline indicates a more important feature in governing the LCZ classification accuracy. Figure 13 shows the overall magnitude of F1-score and AUROC decline from both the CNN and RF classifier and building height datasets (BH_LD and BH_C). The results from both building height datasets did not show significant differences in both classifiers and evaluation metrics. Furthermore, the comparison between F1-score and AUROC metrics decline also shows similar results.

**Fig. 17:**
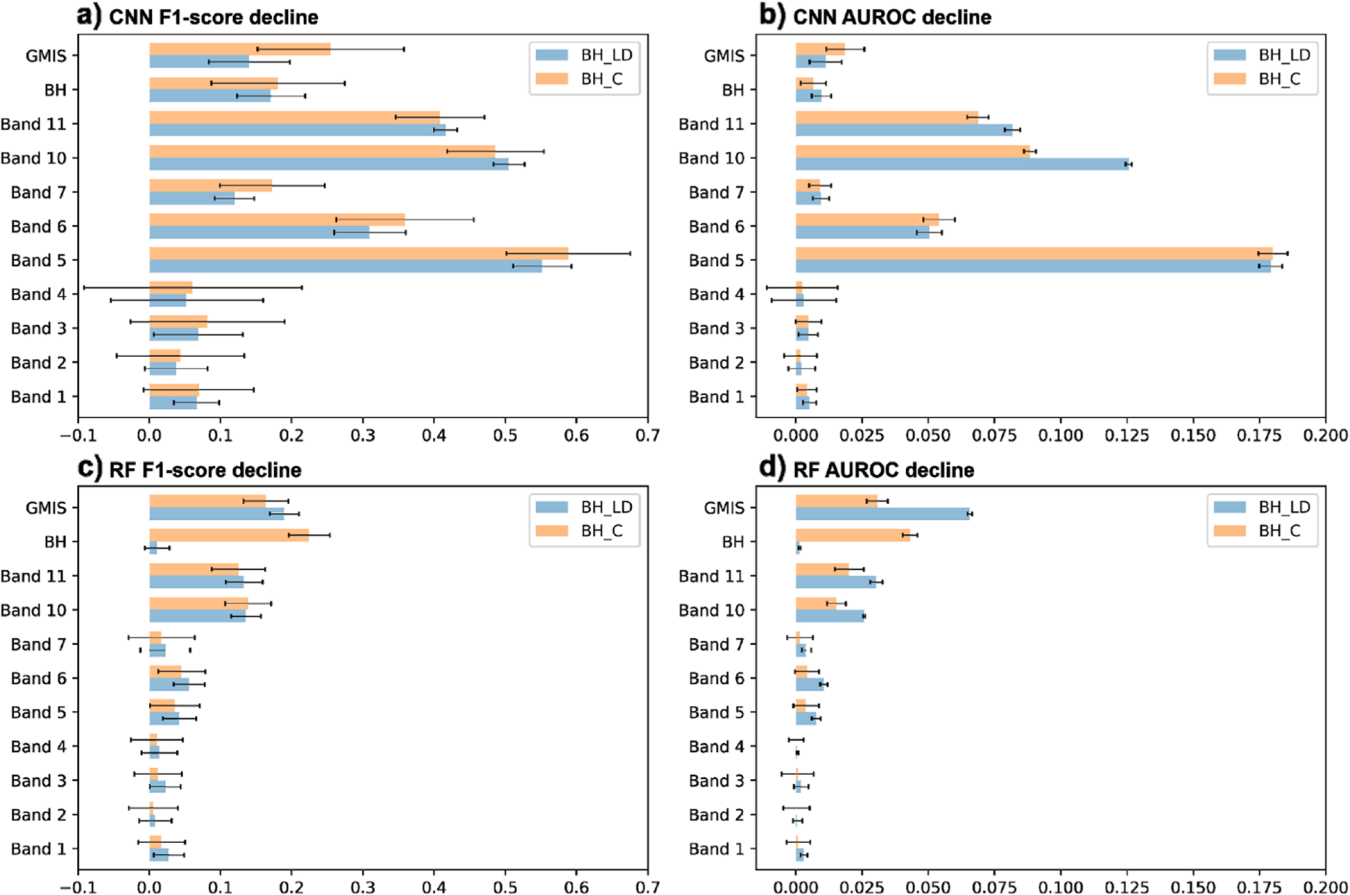
The F1-score decline (**a**, **c**) and AUROC decline (**b**, **d**) for the CNN (**a**, **b**) and RF (**c**, **d**) algorithm from the single-pass forward permutation test. Orange bars show the average decline from BH_C and blue bars from BH_LH. Error bars denoted the minimum and maximum magnitude of decline from all the 20 cross-validation results

In the CNN classifier (Fig. 17a and b), Band 5 (near-infrared) dominate the metrics decline, followed by Band 10 and 11 (thermal infrared). Building height and imperviousness rank after Band 6. This indicates that the characteristic temperature pattern from different urban land uses governs more than the building height and impervious coverage pattern in the CNN classification results. Building height and imperviousness dominate the metrics decline in the RF classifier (Fig. 17c and d), followed by Band 10 and 11. This highlights that building height and imperviousness have a strong influence on improving the LCZ classification results in the RF algorithm.

Comparison between the CNN and RF classifiers show that the overall magnitude decline in CNN is higher than RF. This indicates that the RF is more stable than the CNN. The number of samples difference could contribute to this. Both classifiers use the same pixels for training and testing. However, CNN required samples to be grouped into 5 × 5 pixels, which decreased the number of samples by ~25 times.

## Conclusion

This study used a hybrid framework that ingests urban GIS datasets and remote sensing to systematically compare and evaluate different machine learning classifiers and urban auxiliary datasets. The CNN classifier has the relatively best performance among different classifiers, but the product has a coarser spatial resolution and requires a larger sampling size. In our study, the CNN classifier produces the LCZ map that has 25-times coarser spatial resolution than the other classifiers. This study also evaluated the best classifier using single-pixel input, the random forest with 100 ensemble trees.

Our results show that the building height dataset can substantially enhance the classification performance of mid-rise and high-rise classes. At the same time, the imperviousness data can improve the low-rise classes in the random forest classifier. Furthermore, the single-pass forward permutation test shows that building height and imperviousness are the most critical features in determining LCZ classes. Removal of these two features results in the most significant decline in F1-score and AUROC.

Lidar-derived building height dataset may not be available to every city. Therefore, a satellite-derived building height dataset was also evaluated. The US national categorical mapping of building heights dataset, which is available throughout the continental US, also shows the ability to improve LCZ classification performance. Even though the US national categorical mapping of building height is only available in the continental US, the WUDAPT framework can be easily improved by including the imperviousness data.

The study can serve as a guide for advancing the WUDAPT classification framework by using CNN and/or including urban auxiliary datasets. This framework can be applied to different cities for generating LCZ maps for urban models.

## Data Availability

The Lidar derived building height data are available from City of Austin GIS Open Data Portal (https://austintexas.app.box.com/s/8ah8itbha7u6lis9eipypnz5ljvwta4t). The Landsat 8 data that support the findings of this study are available from USGS EarthExplorer (https://earthexplorer.usgs.gov/). The US national categorical mapping of building heights data are available from USGS Data Release Products (https://www.sciencebase.gov/catalog/item/5775469ce4b07dd077c7088a). The Global Man-made Impervious Surface data are available from the Socioeconomic Data and Applications Center (https://sedac.ciesin.columbia.edu/data/set/ulandsat-gmis-v1). The National Land Cover Database Imperviousness data are available from the Multi-Resolution Land Characteristics consortium (https://www.mrlc.gov/data/nlcd-2019-percent-developed-imperviousness-conus).
